# Integrating traditional practices, livelihoods, and conservation with Indigenous‐led furbearer camera trapping

**DOI:** 10.1111/cobi.70290

**Published:** 2026-04-19

**Authors:** Kathleen A. Carroll, Fabian Grey, Nicholas Anderson, Nelson Anderson, Jason T. Fisher

**Affiliations:** ^1^ School of Environmental Studies University of Victoria Victoria British Columbia Canada; ^2^ Whitefish Lake First Nation #459 Atikameg Alberta Canada

**Keywords:** boreal, Canada, fisher, Indigenous knowledge, Indigenous livelihoods, lynx, marten, red fox, resource extraction, boreal, Canadá, conocimiento tradicional, extracción de recursos, lince, marta, marta pescadora, sustento tradicional, zorro rojo

## Abstract

Contemporary conservation goals have a greater chance of success when practitioners collaborate with Indigenous communities. The importance of such collaborations has spurred calls by Western and Indigenous researchers to engage in equitable coproduction of ecological research that integrates multiple ways of knowing. However, Indigenous‐led conservation research is exceedingly rare. We conducted a case study, applicable globally, in which an Indigenous community experiencing intense industrial resource extraction used Indigenous knowledge and Western science to examine the impacts on species highly valued by the community, namely, furbearing mesocarnivores. Unique knowledge offered by elders and the community suggested rapid declines in many formerly abundant species. Together, we used camera trapping and statistical modeling to investigate the relative abundance of and response to disturbance in lynx (*Lynx canadensis*), red fox (*Vulpes vulpes*), marten (*Martes americana*), and fisher (*Pekania pennanti*) in north‐central Alberta. Low species abundance presented analytical challenges, necessitating pooling species. Several industrial features were associated with (small) increased relative abundance of furbearers (β range 0.12 to 0.30), whereas several others were associated with negative abundance of furbearers (β range −0.25 to −0.64). As Indigenous communities globally face such industrial pressures, we illustrate how Indigenous‐led research design, hypotheses, data collection, and interpretation, coupled with statistical ecological data analysis, can inform pressing conservation decisions. Indigenous Peoples face ongoing challenges accessing essential traditional resources because declining ecosystem functioning is directly linked to Indigenous well‐being. Overall, successful conservation and stewardship are often best achieved through such Indigenous‐led partnerships that address the joint goals of Indigenous livelihoods and conservation.

## Abstract (es)

Integración de las prácticas, los sustentos y la conservación tradicional con el fototrampeo de animales de peletería conducido por indígenas

**Resumen:**

Los objetivos de conservación actuales tienen más posibilidades de éxito cuando los profesionales colaboran con las comunidades indígenas. La importancia de estas colaboraciones ha impulsado a investigadores no indígenas e indígenas a hacer un llamado a la coproducción equitativa de la investigación ecológica (que integre múltiples formas de conocimiento). Sin embargo, la investigación en materia de conservación llevada por indígenas es extremadamente escasa. Realizamos un estudio de caso, aplicable a nivel mundial, en el que una comunidad indígena que sufre una intensa extracción industrial de recursos utilizó el conocimiento indígena y la ciencia no indígena para analizar los impactos sobre especies muy valoradas por la comunidad, es decir, los mesocarnívoros de peletería. El conocimiento único aportado por los ancianos y la comunidad sugirió un rápido declive de muchas especies que antes eran abundantes. Utilizamos cámaras trampa y modelos estadísticos para investigar la abundancia relativa y la respuesta a las perturbaciones del lince (*Lynx canadensis*), el zorro rojo (*Vulpes vulpes*), la marta (*Martes americana*) y la marta pescadora (*Pekania pennanti*) en el centro‐norte de Alberta. La escasa abundancia de las especies planteó dificultades analíticas, lo que obligó a agruparlas. Varias características industriales se asociaron con un (pequeño) aumento de la abundancia relativa de los animales de peletería (rango de β de 0.12 a 0.30), mientras que otras se asociaron con una abundancia negativa de estos animales (rango de β de ‐0.25 a ‐0.64). Dado que las comunidades indígenas de todo el mundo se enfrentan a estas presiones industriales, ilustramos cómo el diseño de la investigación, las hipótesis, la recopilación de datos y la interpretación, dirigidos por los indígenas, junto con el análisis estadístico de los datos ecológicos, pueden servir de base para tomar decisiones urgentes en materia de conservación. Los pueblos indígenas se enfrentan a retos constantes para acceder a recursos tradicionales esenciales, ya que el deterioro del funcionamiento de los ecosistemas está directamente relacionado con su bienestar. En general, la conservación y la gestión responsables suelen lograrse mejor a través de estas colaboraciones dirigidas por los indígenas que abordan los objetivos conjuntos de los medios de vida indígenas y la conservación.

## INTRODUCTION

Indigenous communities’ livelihoods and wildlife conservation are mutually interdependent, embodying reciprocal biocultural interactions (Zent & Zent, 2023). However, the rights of Indigenous peoples to use culturally important resources such as wild game, fish, and native plant species have historically been restricted by government policies, natural resource laws, or the rights of resource developers (Bruce et al., 2024). Accordingly, in 2013, Article 11 of the United Nations Declaration on the Rights of Indigenous Peoples (UNDRIP) recognized the right of Indigenous Peoples to practice and revitalize their cultural traditions (United Nations, 2007). Likewise, Article 8 of the Convention on Biological Diversity mandates nations to “respect, preserve and maintain knowledge, innovations and practices of indigenous and local communities embodying traditional lifestyles relevant for the conservation and sustainable use of biological diversity…” (Secretariat for the Convention on Biological Diversity, 2011). Despite these mandates and the growing recognition that Indigenous Peoples’ lands are vital to biodiversity conservation (Schuster et al., 2019), the expanding cumulative footprint of human activity in natural landscapes negatively impacts wildlife communities on traditional territories (Fisher et al., 2021). Concomitant with many calls from Western scientists (Vallet et al., 2023) for research equity, which embodies the opportunity for everyone to reach full potential, acknowledging that different groups may require different levels of support to achieve this, Indigenous participation in research is growing (Straka et al., 2018; Ward & Gorelick, 2018). However, Indigenous‐led research in conservation has been rare, with Artelle et al. (2021) and Housty et al. (2014) from Canada, and participatory or collaborative action research from Latin American countries (e.g., Athayde et al., 2014; Fals Borda, 1987) providing excellent early examples.

In western Canada, many Indigenous Peoples live under Treaty 8 (Fumoleau, 2004; Queen's Printer for Canada, 1966), which guarantees Indigenous rights to meaningful access to hunting, trapping, and gathering of traditional resources. *Furbearer* is herein defined as a mammalian mesocarnivore allowed under Canadian law to be harvested via trapping, which is an expression of Indigenous culture, way of life and tradition, a source of income, as well as a focus for resource management and conservation (Collins & Murtha, 2009; Gombay, 2025; Passelac‐Ross, 2005). Furbearers are a subject of conservation concern worldwide (Marneweck et al., 2021), including western Canada, where forest‐specialist or forest‐affiliated species are of particular concern. Across the West, lynx (*Lynx canadensis*) populations are declining as development increases (Bayne et al., 2008). Red fox (*Vulpes vulpes*), marten (*Martes americana*), and fisher (*Pekania pennanti*) decline in relative abundance with increasing landscape development in mountain (Heim et al., 2019) and boreal systems (Fisher & Burton, 2018; Stewart et al., 2019). Climate change is also implicated in species like marten, fishers, and wolverines (*Gulo gulo*), as changing snow conditions alter competitive dynamics (Fisher et al., 2022; Pauli et al., 2022). The conservation of furbearers is an emerging focus for many North American ecosystems, and furbearer declines impact Indigenous Peoples’ culture and well being (Muir, 2022; Priadka et al., 2022).

The first step in furbearer conservation is evaluating the magnitude and causes of population declines. The long‐term local knowledge of Indigenous People is well‐suited to identify sources of furbearer decline, but it often does not carry the weight of published science in the eyes of industry and government. Indigenous‐led science (Kimmerer & Artelle, 2024) in which communities lead, not just participate in science, is well situated to link these knowledge systems but remains exceedingly rare. Integrating Western and Indigenous knowledge informs both trends and causes. Science from this perspective can generate more insightful research hypotheses, leading to better research outcomes and empowering communities (Adams et al., 2014; Colbourne et al., 2019; Mistry & Berardi, 2016).

Herein, we present a case study that transitions from traditional Indigenous knowledge to Indigenous‐led research, incorporating elements of Western science, to address and highlight conservation issues within a traditional territory. Elders and the Cree community of Whitefish Lake First Nation (WLFN), who live and trap this land and carry ancestral knowledge, noted that furbearers, particularly those associated with mature boreal forests, had been declining in concert with the rise of resource extraction. The western Nearctic boreal forest has borne the effects of extensive forest harvesting, petroleum extraction, and transportation infrastructure (Pickell et al., 2013, 2015), and the WLFN territory is no exception (Fisher et al., 2021). The first published research by an Indigenous community on the effects of oil sands and timber harvesting on wildlife (Fisher et al., 2021) brought statistical insight to a problem that elders and community members had long maintained: the mammal community is shifting in response to resource extraction. However, deeper latent problems related to furbearers remained.

**FIGURE 1:**
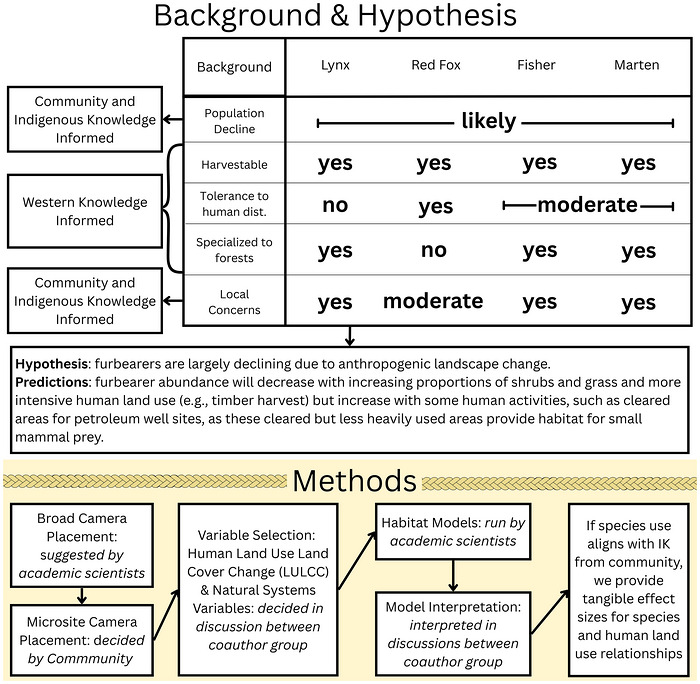
Derivation of current species knowledge relative to the study of furbearers (i.e., lynx, red fox, fisher, and marten) and the workflow used.

The WLFN's observations and historical knowledge passed to the Indigenous scientist coauthors through conversations with their families and friends informed our research hypothesis about furbearer declines (Figure 1) that the Indigenous scientist coauthors finalized. Indigenous scientists hypothesized that furbearers were largely declining due to anthropogenic landscape change. Mature forest loss that began after wildfires in the last decade was exacerbated by subsequent forest harvest and petroleum extraction. These activities replace old forests with young shrubs and saplings, providing substantial, spatially clustered forage for herbivores (Fisher & Wilkinson, 2005). The dominant carnivore, wolf (*Canis lupus*), has benefited from disturbance and herbivore subsidies via well‐established ecological mechanisms (Dickie et al., 2017; Latham et al., 2011; Serrouya et al., 2017; Wasser et al., 2011). Aside from a few community‐scale analyses (Curveira‐Santos et al., 2024; Fisher & Burton, 2018; Wittische et al., 2021), the result of shifting top‐down and bottom‐up effects on midtrophic mesocarnivores remains (to Western science) unknown. To quantify these observations, we used wildlife camera trapping (Burton et al., 2015; Steenweg et al., 2017) as a monitoring tool to sample furbearing forest carnivore relative abundance.

Based on conversations between the WLFN coauthors and elders and the community of WLFN, as well as a shared understanding of furbearer ecology, we expected to see a lower abundance of furbearers, including lynx, fisher, marten, and red fox, associated with increasing proportions of shrubs and grass and more intensive human land use (e.g., timber harvest). We also expected that some human activities, such as cleared areas for petroleum well sites, may increase furbearing forest carnivore species’ use, as these cleared but less heavily used areas provide habitat for small mammal prey. We also anticipated having few data points for each species, given WLFN's trapping experience, which suggests there are few remaining individuals of each species in their traplines.

F.G., N.A., and N.A. are members of the WLFN in Alberta, Canada. We gathered information from friends, family, and community members who hunt, fish, trap, and travel the land about the state of wildlife on their territory, and garnered funding for and executed this research. This article is based entirely on their views and ideas. K.A.C. and J.T.F. are academic scientists who served as statistical analysts and scientific writers. F.G., N.A., and N.A. provided oversight and editing for the analyses and writing. The data and ideas herein are the property of the Indigenous scientists.

## METHODS

### Study area

Our study area encompassed the land on which WLFN has lived for millennia in north‐central Alberta, Canada (Appendix S1). It was dominated by central mixed‐wood forests interspersed with many small lakes, bogs, wetlands, fescue grasslands, both open and closed conifer stands, and closed shrublands (Alberta Biodiversity Monitoring Institute [ABMI]). Recent oil and gas extraction and forestry, which we refer to as *anthropogenic features*, spanned the landscape. This disturbance included petroleum exploration seismic lines (Dabros et al., 2018, 2022), vehicle‐accessible roads, trails accessible by off‐highway vehicles, pipelines, exploration and drilling well pads, and forest harvest cut blocks (Fisher & Burton, 2018; Fisher et al., 2021). Elders noted that the changes wrought by forest harvest, subsequent silviculture, and petroleum extraction have altered the abundance and distribution of plant and animal communities, observations now supported by Indigenous‐led science (Carroll, Grey, Anderson, Anderson, & Fisher, 2025; Carroll, Grey, Anderson, Anderson, Goward, et al., 2025). They noted that many species, including moose and those traditionally harvested for fur (i.e., lynx, fox, fisher, marten), were less common near WLFN, posing community concerns about traditionally relied‐upon resources. This ecosystem also experienced several severe fire years.

### Camera trapping

The Indigenous scientists and coauthors invited non‐Indigenous scientists to codesign the research study to capture medium‐ and large‐mammal data (following Fisher et al. [2021] and Carroll, Grey, Anderson, Anderson, & Fisher [2025]). We first stratified the study area into four strata (mesic conifer, hygric conifer, mesic deciduous, hygric deciduous) and overlaid a 2‐km^2^ grid‐cell mesh on it. From each stratum, we randomly drew cells for sampling, discarding those without access. Within these cells, the community scientists selected deployment sites for cameras on active wildlife trails. They deployed 130 Reconyx Hyperfire 2 (Holmen) cameras between 2018 and 2023 (Appendix S2). The community deployed 75 active cameras from December 2018 to April and May 2019, and added 25 in March 2019. All 100 cameras were active until November 2019. WLFN deployed an additional 30 cameras from June 2022 to July 2023. All cameras were placed approximately 1.5 m above ground on active wildlife trails, and camera sensors were set to high sensitivity, with no programmed delays between photographs, adopting techniques used by Fisher and Burton (2018). WLFN staff and volunteers used TimeLapse2 Image Analysis software to classify images to species, and image tagging accuracy was quantified using random double‐observer checks. Data and images were retrievable from 121 cameras (96 of the first 100; 21 of the 30). Images were grouped by monthly sampling periods.

### Habitat model

We identified 30 land‐cover, human features (or footprint), and fire‐related predictor variables of interest to WLFN or related to furbearer ecology (Table 1). We estimated the monthly presence and absence of each species across cameras and controlled for the number of days each camera was functional. In this approach, we assumed that if a furbearer was not detected at a site during sampling, we could reliably state it did not occur there, rather than assuming false absence as in an occupancy framework (MacKenzie et al., 2003). Thus, our response metric was the number of months that a species was detected or not within the sampling period, with each month considered a Bernoulli trial, commonly called a proportional binomial. We modeled furbearer monthly detections with binomial generalized linear models (GLMs) with 30 land‐cover, human features, and burn area variables, all centered and *z* scaled (Table 1). However, fire was removed due to limited sample size and model convergence concerns. We summarized the area of each response variable across spatial scales by generating 20 concentric circular buffers around each site, ranging from 250‐ to 5000‐m radius at 250‐m intervals (Fisher et al., 2011). Our GLM models were run across all sites with bidirectional stepwise Akaike information criterion (AIC) model selection, which identified the best‐supported model and best‐supported spatial scale. We examined variance inflation factors (<4), variable correlation (Appendix S3), and residual‐versus‐leverage plots. The estimate and standard error were then assessed for each variable in each model.

**TABLE 1:** Predictor variables used to develop models of furbearing forest carnivore selection.

Category	Variable	Description	Source
Land cover	Exposed	Exposed soil	Alberta Satellite Land Cover, Alberta Agriculture and Forestry, Government of Alberta
	Closed deciduous and mixed forest	Closed aspen, balsam poplar, birch, and mixed‐wood forest	
	Grassland	Fescue grassland	
	Water	All water bodies	
	Bogs and wetlands	Graminoid wetlands, shrubby wetlands, undifferentiated wetlands, and black spruce bogs	
	Open conifer forest	Open undifferentiated coniferous forests	
	Closed shrubland	Closed upland shrub	
	Closed conifer forest	Close pine, closed Engelmann or white spruce, and closed undifferentiated conifers	
Human features	Transmission lines	Cleared corridors designated for the location of power transmission line infrastructure	Alberta Biodiversity Monitoring Institute Wall‐to‐Wall Human Footprint Inventory (2021)
	Borrow pits	Excavation outside the road right‐of‐way done solely to remove or provide borrowed material for subbase construction for a specific roadway project; includes any other associated infrastructure, such as access roads	
	Clearing	Human footprint features related to various industrial activities	
	Cultivation	Lands where the forest or shrubs have been removed to plant crops or grass species for livestock grazing	
	Facilities	Human footprint features related to various industrial activities	
	Mines	Human footprint features directly related to mining activities	
	Trails	Cleared corridors surfaced with dirt or low vegetation for human or vehicle access	
	Vegetated edges	Disturbed vegetation alongside road edges and railway edges, including ditches and other industrial features	
	Wells	Ground cleared for an oil or gas well pad where at least one well is currently active	
	Harvest	Areas where forestry operations have occurred (clearcut, selective harvest, salvage logging, etc.)	
	Recreation	Human footprint related to vegetated facilities and recreation	
	Residential	Residential developments with buildings for human habitation	
	Seismic	Cleared corridors created during hydrocarbon exploration	
	Seismic 3D	Cleared corridors created during hydrocarbon exploration	
	Pipeline	Line of underground and overground pipes of substantial length and capacity used to convey petrochemicals; physical clearing contains underground and aboveground high‐pressure pipelines	
	Roads	Nonvegetated, impermeable surfaces used for motorized vehicle or aircraft transportation or access	
Fire	Area burned (0–5 years)	Area burned from 2019 to 2023	Fire perimeter data, Wildfire Management Branch, Government of Alberta
	Area burned (6–10 years)	Area burned from 2014 to 2018	
	Area burned (11–15 years)	Area burned from 2009 to 2013	
	Area burned (16–20 years)	Area burned from 2004 to 2008	
	Area burned (21–25 years)	Area burned from 1999 to 2003	
	Area burned (26–29 years)	Area burned from 1995 to 1998 (shorter was based on lack of 1994 burn area data)	

## RESULTS

### Furbearer detections

There were very few detections of furbearer species. Across the entire study period (2018–2023), fishers were only detected 69 times, lynx 172 times, marten 17 times, and red fox 10 times (based on unique site‐by‐day combinations). In comparison, many other species were detected thousands (e.g., black bears [*Ursus americanus*] >3200 and moose [*Alces alces*] >9400) or tens of thousands of times (e.g., white‐tailed deer [*Odocoileus virginianus*] >13,800). Moreover, these detection rates are much lower than those reported in other studies that used the same camera deployment protocols (Beirne et al., 2021; Fisher & Burton, 2018) in the Oil Sands Monitoring Program (Roberts et al., 2022). The unexpectedly low detection rates of furbearers made it impossible to generate species‐level models and instead necessitated a single model that pooled all species; thus, the interpretation of model estimates is that of a common statistical response across all furbearers detected, limiting species‐specific inferences.

### Habitat model

**FIGURE 2:**
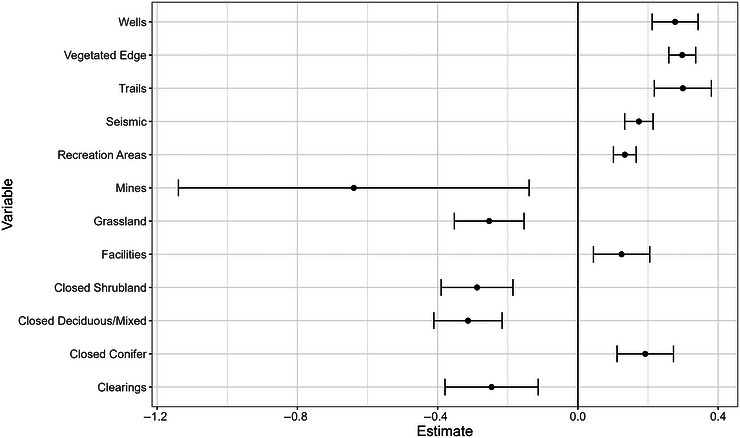
Effect size and direction of predictor variables on furbearer selection (error bars, GLM model standard errors).

Multiple landscape features associated with industrial development were associated with furbearer species on the territory. These features appeared to affect furbearers when accumulated at larger spatial scales: the best‐performing habitat model resolution for furbearers was a 4500‐m radius around each site (ΔAIC = 5.06) (Appendix S4). This model included petroleum well pads, vegetated edges, trails, petroleum exploration seismic lines, recreation areas, mines, facilities, clearings, grasslands, closed shrubland, closed deciduous and mixed wood, and closed conifer. Not all features were associated with lower furbearer abundance: wells, trails, vegetated road edges, seismic lines, recreation areas, facilities, and closed conifer forest all had positive βs, but all effect sizes were small (Appendix S5). The remaining landscape features, including mines, grassland, closed shrubland, closed deciduous and mixed wood, and industrial clearings, had negative βs, with all but mines having effect sizes and confidence intervals less than −0.42. Mines had the largest β but the widest confidence intervals, spanning from nearly −1.2 to <−0.2 (Figure 2). In summary, several industrial features were associated with (small) increased relative abundance of furbearers, whereas several others were associated with negative abundance of furbearers.

Anthropogenic landscape disturbance both positively and negatively impacted furbearer habitat use across WLFN's territory. The WLFN anticipated, and the data confirmed, that furbearers avoided many areas, such as shrublands, grasslands, and areas with intensive human land use—in this case, sites with higher densities of active oil industry facilities (covariate mines) where land is cleared and human activity is preventing regeneration. Statistical models were also concordant with WLFN observations that some industrial features, such as wells, may increase furbearing forest carnivore species’ use, as these regenerating areas (less heavily used, or even unused by humans) provide habitat for small mammal prey (Darling et al., 2019). Furbearer occurrence increased with other regenerating anthropogenic features, including trails, vegetated edges along roads, recreation areas, seismic lines, and grassy‐perimetered facilities, all areas that could potentially facilitate small mammal prey availability, scavenging, or ease of travel (Darling et al., 2019; Fisher & Ladle, 2022). WLFN members also saw these species near local roadways and when monitoring trails, aligning with the model outcomes.

## DISCUSSION

### Implications for conservation on WLFN traditional territory

Furbearers were remarkably rare in this system, with low detection rates and naïve occupancy, concordant with the community and elder observations shared with the Indigenous coauthors. Furbearers displayed varying responses to human land use at larger spatial scales (4500 m) rather than at fine scales. This generates a conundrum: the presence of industrial features is associated with lower abundance of furbearers, but without a clear, substantial effect, while higher presence of industrial features is associated with higher abundance of furbearers.

Why are furbearers declining? Two hypotheses emerge. First, furbearers were intensively harvested post‐European colonization, and the fur trade motivated the occupation of Canada. Collins et al. (2020) showed that the median population of eight furbearer species has declined by 15% since 1850. Poorly regulated harvesting may continue to contribute to declines (Province of Alberta, 2024), coincidentally timed with landscape development. Second, the answer may lie in the selection of anthropogenic features by furbearers. Small mammals (rodents and snowshoe hares) exploit these sites (Darling et al., 2019; Fisher et al., 2021), which are furbearer prey. Disturbed sites are often treated with glyphosate to prevent deciduous regeneration (Carroll, Grey, Anderson, Anderson, Goward, et al., 2025) as well as rodenticide to reduce pests. The latter accumulate to toxic levels in furbearers such as fishers. Indigenous‐partnered research in this same region showed high levels of second‐generation rodenticides (SGARs) in harvested fisher carcasses (Thomas et al., 2017). The population consequences of applying poison to thousands of landscape features have yet to be examined.

Perhaps both mechanisms are occurring. Our results and Indigenous knowledge suggest population declines are real and are in part associated with industrial landscape features. Across Alberta, many furbearers are negatively associated with anthropogenic features (Morehouse et al., 2025), and other communities have reported low furbearer numbers (O'Connor et al., 2022). Thus, effective management of landscape features postdisturbance is needed to maintain furbearer populations and the WLFN's meaningful access to this key cultural resource.

Substantial concerns remain regarding furbearer abundance across WLFN's traditional territory and, more broadly, across North America. WLFN relies on these species for clothing (e.g., winter gloves) and food and uses what is left as bait for other hunting and trapping efforts. Members of the Nation view mesocarnivores as a harbinger of system changes, implying that when one species or group is doing badly, they all are because the system is interconnected. Their concerns extend beyond the observations of human land‐use features outlined here, as contamination, forest fire fuel load (duff), fire, and herbicide and pesticide use are increasing across the landscape. Overall, the few observations of furbearers we found, along with the knowledge that these species are negatively impacted by increasing development (Bayne et al., 2008; Fisher & Burton, 2018; Fisher et al., 2021; Heim et al., 2019; Stewart et al., 2019), and the concerns of community, suggest that the furbearers in WLFN's traditional territories continue to face substantial conservation challenges. Thus, we continue to urge industries and government to engage equitably and responsibly with First Nations and to uphold Indigenous conservation and stewardship in policy.

### Implications for Indigenous communities globally

We provide a case study of how WLFN's Indigenous‐led research tackled a pressing problem of both sovereignty and conservation involving declining furbearer populations needed for cultural and monetary subsistence. The results of this study are, of course, place based, but Indigenous communities are rooted in place, and hence so too will be Indigenous research. Although our focus was on furbearers, the camera‐trap methods used can be employed for any wildlife species of concern, including mammals, fish, birds, or insects (Fisher, 2025). Our emphasis was on the application of technology to Indigenous knowledge and ideas. This fully Indigenous‐led conservation study, from traditional knowledge to research publication, used easily accessible research methods and Western science for statistical analyses, was written jointly, and offers a model for Indigenous communities globally.

Indigenous‐led research is essential for several reasons, including the importance of equitable conservation research (Newing et al., 2024), the benefits of integrating many ways of knowing (Bartlett et al., 2012), and the fact that Indigenous communities are stewards of some of the most important ecologically intact landscapes and forests (Fa et al., 2020; Garnett et al., 2018) containing vast distributions of terrestrial mammals (O'Bryan et al., 2021). The functioning of many landscapes is intrinsically linked to the Indigenous People in that area. Wildlife for peoples’ needs declines as the land and species become increasingly unsustainable. Moreover, Indigenous knowledge and research are especially important when they are used in court cases (Hamilton & Ettinger, 2023) or government‐to‐government negotiations in support of Human or Treaty Rights.

Much has been written about the importance of integrating Indigenous knowledge and Western science (Abu et al., 2020; Colbourne et al., 2019; Popp et al., 2019), but concrete examples are only recently emerging in Canada (Adams et al., 2014; Fisher et al., 2019; Service et al., 2014). However, Indigenous representation is growing globally, both as Indigenous‐led research and in traditional Western academic settings, where the importance and value of principal investigator (PI) diversity are increasingly recognized. We found that Indigenous‐led research can be informed by local and historical knowledge and executed using statistical designs and analysis to inform and empower the management of Indigenous resources on Indigenous lands. We also highlight the many spheres of Indigenous identity to which an individual may adhere because the Indigenous coauthors here served as community voices, Indigenous knowledge holders, and scientists.

## AUTHOR CONTRIBUTIONS

**Kathleen A. Carroll**: Conceptualization of analyses; formal analyses; visualization; writing—original draft. **Fabian Grey**: Conceptualization of surveys; data curation; writing—original draft. **Nicholas Anderson**: Conceptualization of surveys; data curation; writing—review and editing. **Nelson Anderson**: Conceptualization of surveys; data curation; writing—review and editing. **Jason T. Fisher**: Conceptualization of analyses; supervision; writing—original draft.

## Supporting information



Supporting Information

## Data Availability

All relevant data are cited or are property of Whitefish Lake First Nation and other First Nations of Canada (see the CARE Principles for Indigenous Data Governance: https://www.gida‐global.org/care). Access to these datasets can be obtained by contacting F.G.
