# Supplementary material for: Integrating traditional practices, livelihoods, and conservation with Indigenous‐led furbearer camera trapping
Source: Conserv Biol. 2026 Apr 19;40(4):e70290. doi: 10.1111/cobi.70290 (PMC13392795; doi:10.1111/cobi.70290)
Supplement: Supplementary file 1 — Supporting Information [file COBI-40-e70290-s001.pdf]

Integrating traditional practices, livelihoods, and conservation with Indigenous-led furbearer camera trapping

Supplemental File

Kathleen A. Carroll[1,2*](ORCID: 0000-0003-3853-0501), Fabian Grey[2], Nicholas Anderson[3], Nelson Anderson[3], Jason T. Fisher[1](ORCID: 0000-0002-9020-6509)

[1] University of Victoria, School of Environmental Studies, PO, Box 1700 STN CSC Victoria, BC V8W 2Y2, Canada

[2] Current Affiliation: Quest Lab, Department of Natural Resources Science, University of Rhode Island, Kingston, RI 02881, USA.

[3] Whitefish Lake First Nation #459. General Delivery, Atikameg, AB T0G 0C0, Canada

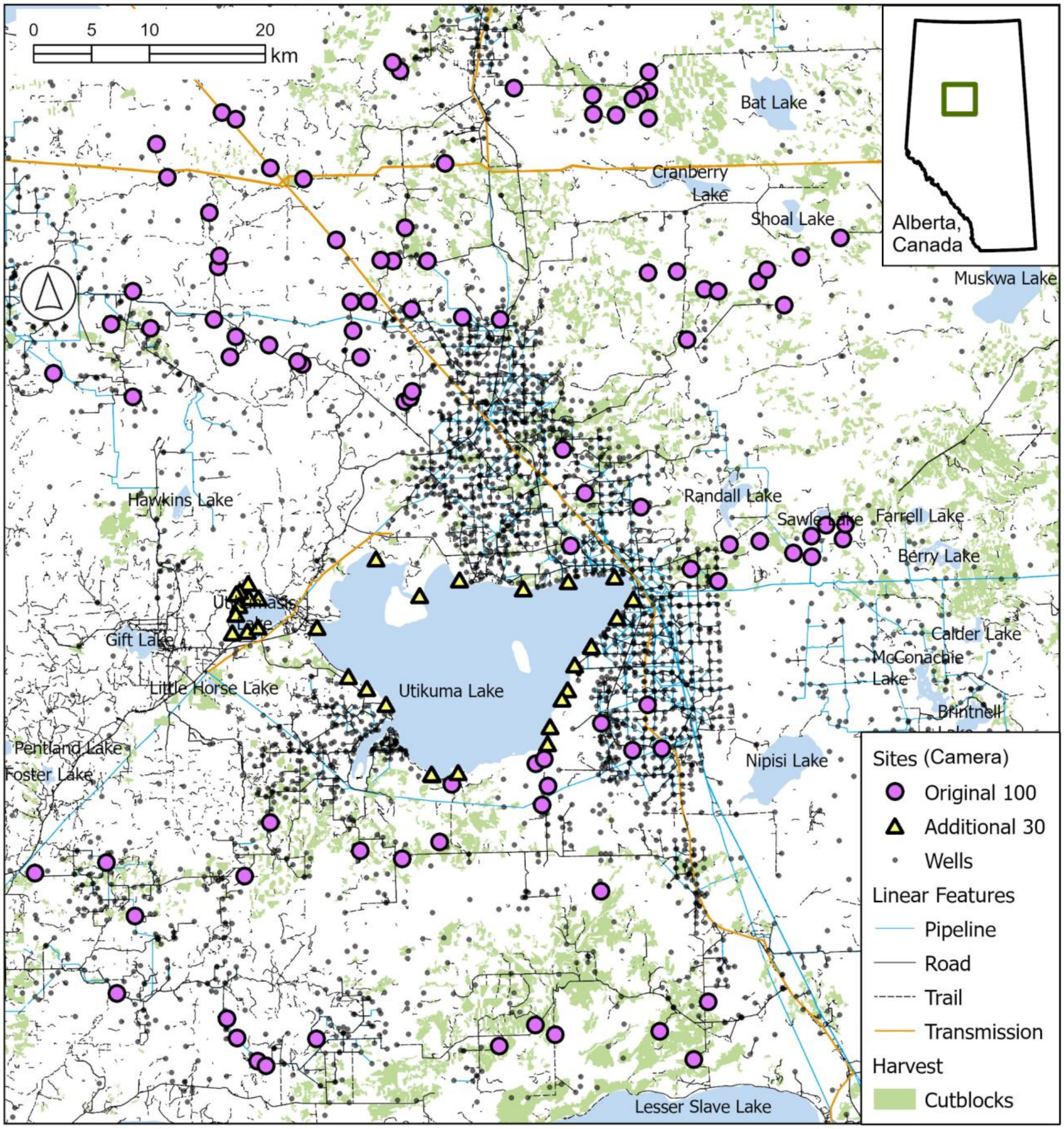

S1. The study area, including Whitefish Lake First Nation's Traditional Territory, anthropogenic features, and locations of remote trail cameras used to photograph wildlife. The original 100 (purple circles) sites refer to cameras placed before 2020, and the additional 30 sites (yellow triangles) represent the cameras added after 2020.

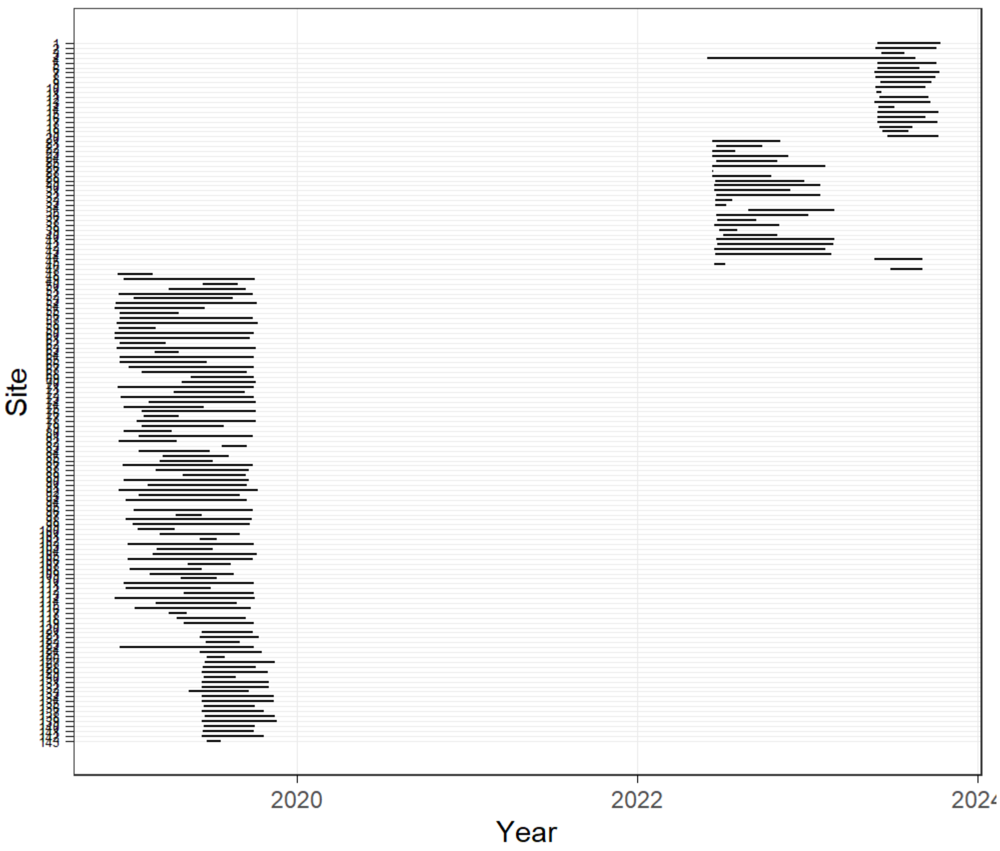

S2. Temporal camera deployment data. There is no overlap between forest sites (pre-2020) and lake sites (post-2022).

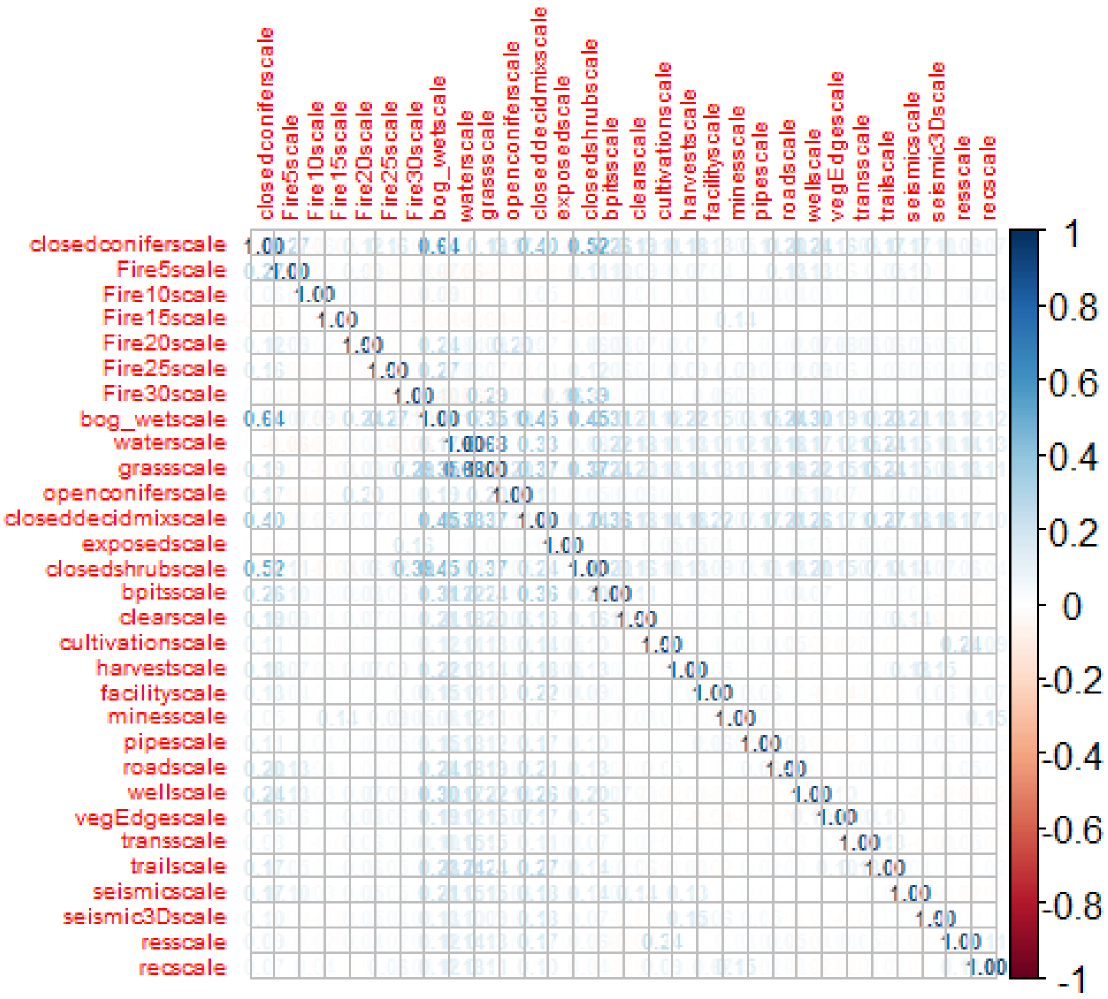

S3. Correlation between scaled predictor variables, none of which were correlated above a threshold of 0.70.

S4. Top model across spatial scale, sample size (n), AIC values (including delta [Δ] AIC and weight), the deviance (D^2), and adjusted deviance explained by the model at each scale.

Scale (m)	n	AIC	ΔAIC	AIC Weight	D^2	aD^2
250	1003	773.13	102.07	0.00	7.26	0.07
500	1003	727.50	56.45	0.00	14.46	0.14
750	1003	764.51	93.46	0.00	8.93	0.08
1000	1003	730.23	59.18	0.00	14.66	0.14
1250	1003	723.99	52.94	0.00	17.23	0.16
1500	1003	755.89	84.83	0.00	10.93	0.10
1750	1003	771.71	100.66	0.00	7.79	0.07
2000	1003	725.54	54.49	0.00	15.40	0.15
2250	1003	722.21	51.16	0.00	16.56	0.16
2500	1003	758.97	87.92	0.00	9.81	0.09
2750	1003	752.92	81.86	0.00	10.45	0.10
3000	1003	721.84	50.79	0.00	16.94	0.16
3250	1003	721.01	49.95	0.00	16.44	0.16
3500	1003	763.09	92.04	0.00	9.47	0.09
3750	1003	750.62	79.56	0.00	11.44	0.11
4000	1003	708.97	37.92	0.00	18.34	0.18
4250	1003	743.25	72.20	0.00	12.61	0.12
4500	1003	671.05	0.00	1.00	25.27	0.24
4750	1003	719.19	48.14	0.00	17.67	0.17
5000	1003	748.86	77.80	0.00	12.35	0.11

S5. Model estimates from the top-performing model of furbearer selection with a 4500 m buffer.

Variable	B (Beta)	Error
Closed Conifer	0.1922	0.08047
Grassland	-0.25289	0.09942
Closed Deciduous/Mixed	-0.31347	0.09747
Closed Shrubland	-0.28757	0.10242
Clearings	-0.2461	0.13294
Facilities	0.12462	0.08026
Mines	-0.63918	0.50017
Wells	0.27735	0.06556
Vegetated Edge	0.29766	0.03829
Trails	0.29943	0.08136
Seismic	0.17394	0.04057
Recreation Areas	0.13385	0.03246

Additional Papers from the Global South from Reviewer # 3

Fals Borda, O., Bonilla, V., & Castillo, G. 1972 *Causa popular, ciencia popular*. Bogotá: Publicaciones de La Rosca.

Fals Borda, O. 1973 Reflexiones sobre la aplicación del método de estudio acción en Colombia. *Revista Paraguaya de Sociología*, 10(26), 25-36.

Fals Borda, O. 1985 *Conocimiento y poder popular*. Bogotá: Siglo XXI.

Fals Borda, O. 1987 The Application of Participatory Action-Research in Latin America. *International Sociology*, 2(4), 329–347. https://doi.org/10.1177/026858098700200401 There are many more literary references written by Fals-Borda but those are enough to illustrate my point.

Most of the Collaborative or Participatory Action Research projects in Latin American countries is never published since they are alive processes and often times is even safer and convenient to keep the information low-profile. However, a *very* superficial sample of publications associated to PAR/Car (worldwide) could be:

Athayde, S., Bartels, W.-L., Buschbacher, R., & Rosa, R. D. 2014 Aprendizagem colaborativa, transdisciplinaridade e gestão socioambiental na Amazônia: abordagens para a construção de conhecimento entre academia e sociedade. *Revista Brasileira de PósGraduação : RBPG*, 10(21). https://doi.org/10.21713/2358-2332.2013.v10.419

Carter, J. 1996. Recent Approaches to Participatory Forest Resource Assessment. *Rural Development Forestry Study Guide* N° 2. Overseas Oxford, LTK, Development Institute. UK. 322 p.

Chambers, R. 1983. *Rural Development; Putting the Last First*. Logman, London.

Cornish, F., Breton, N., Moreno-Tabarez, U., Delgado, J., Rua, M., de-Graft Aikins, A., &

Hodgetts, D. 2023 Participatory action research. *Nature Reviews Methods Primers*, 3(1),

Article 34. https://doi.org/10.1038/s43586-023-00214-1 *although did not recognize the Latin American tradition*

Czerwenka, J. y Gudynas, E. 2001. Las múltiples caras de la participación social en las áreas protegidas. *Fundamentos de Conservación Biológica. Perspectivas latinoamericanas*. En: Primack, R. Rozzi, R., Feinsinger, P., Dirzo, R. y Massardo, F. Fondo de Cultura Económica: 509-510. México.

Danielsen, F., Burges, D.N., Balmford, P., Donald, F.P., Funder, M., Jones, J.P.G., Alviola, P., Balete, D.S., Blomley, T., Brashares, J., Child, B., Engho:, M., Fjeldså, J., Holt, S., Húbertz, H., Jensen, A.E., Jensen, P.M., Massao, J., Mendoza, M.M., Ngaga, Y., Poulsen, M.K., Rueda, R., Sam, M., Shielboe, T., Stuart-Hill, G., Topp-Jorgensen, E. y Yonten, A. D. 2008.

Local Participation in Natural Resource Monitoring: a Characterization of Approaches. *Conservation Biology*. 23: 31-42.

Estrada Martines E & Alvaro Sepulveda Varon 2024 Etnobiología Comprometida. Encuentros, acuerdos, producción de conocimientos y acciones colectivas entre pueblos, territorios y academia. El Volcán Insurgente.

https://www.enelvolcan.com/enefebmarabrmayjun2024/810-etnobiologiacomprometida-encuentros-acuerdos-produccion-de-conocimientos-y-accionescolectivas-entre-pueblos-territorios-y-academia

Evans K., de Jong W., Cronkleton, P., Sheil, D., Lynam, T., Kusumanto, T. y Colfer, C.J.P. 2006. *Guide to Participatory Tools for Forest Communities*. Center for International Forestry Research (CIFOR). Bogor, Indonesia.

Fernández-Giménez, M.E., Ballard, H.L. y Sturtevan, V.E. 2008. Adaptative management and social learning in collaborative and community-based monitoring: a study of five community based forestry organizations in the western USA. *Ecology and Society* 13(2):4 Franquesa-Soler M, Jorge Sales L, Silva-Silva Rivera E. 2023 Participatory action research for primate conservation: A critical analysis of a nonformal education program in Southern Mexico. *Am J Primatol*.85(5):e23450. doi: 10.1002/ajp.23450. Epub 2022 Nov 1. PMID: 36317585.

Goodnough, K. 2011 *Taking Action in Science Classrooms through Collaborative Action Research*. Sense Publishers. https://doi.org/10.1007/978-94-6091-583-3

Holt-Giménez E. 2006. *Campesino a Campesino: Voices from Latin America's Farmer Movement for sustainable Agriculture*. Food First Books, Oakland, CA. USA.

Holland, J. 1998. *Who changes? Institutionalizing Participation in Development*. Intermediate Technology Publications, Ltd. London, UK.

Hunn, E., Johnson, D., Russell, P., & Thornton, T. 2003 Huna Tlingit Traditional Environmental

Knowledge, Conservation, and the Management of a "Wilderness" Park1. *Current*

Anthropology, 44(S5), S79–S103. https://doi.org/10.1086/377666

La Torre-Cuadros M.A. 2011. *Catálogo Arte Kakataibo. Comunidades Nativas Yamino y Mariscal Cáceres*. World Agroforestry Centre (ICRAF). Lima. 52 pp.

La Torre-Cuadros M.A., Arnillas M.C., Arellano G.M.A. 2012. Fortalecimiento de capacidades para la gestión del Santuario Nacional Pampa Hermosa: Construyendo las bases para un manejo adaptativo para el desarrollo local. Memoria del proyecto. *ICRAF Working Paper* N° 141. World Agroforestry Centre (ICRAF) y Center for Biodiversity and Conservation

(CBC) at the American Museum of Natural History, New York. Lima, Perú. http://dx.doi.org/10.5716/WP12005.pdf

Méndez, V., Caswell, M., Gliessman, S., & Cohen, R. 2017 Integrating Agroecology and Participatory Action Research (PAR): Lessons from Central America. *Sustainability*, 9(5), 705. https://doi.org/10.3390/su9050705

Murphree, M.W. 1996. “Ex Africa semper aliquid novi?” Considerations in Linking Environmental Scholarship, Policy and Practice. *Pan African Symposium on the Sustainable Use of Natural Resources and Community Participation*. IUCN and IFRA. Harare, Zimbabwe.

Perkins, P. E. 2023 *Climate justice and participatory research : building climate-resilient commons* (1st ed.). LCR Publishing Services. https://doi.org/10.1515/9781773854090 Rappaport, J., & Ramos Pacho, A. 2005 Una historia colaborativa:retos para el diálogo indígena-académico. *Historia crítica* (Bogotá, Colombia), 29, 39-62. https://doi.org/10.7440/histcrit29.2005.02

Rappaport, J., Flórez G., L., & Pérez, P. 2024 *Historieta doble : a graphic history of participatory action research*. University of Toronto Press.

Pataxó Hãhãhãi, H. D. S., & Rodrigues, E. 2025 Participatory Ethnobotany in indigenous health: study conducted by a Pataxó Hãhãhãi ethnobotanist among his people, Brazil.

Journal of Ethnobiology and Ethnomedicine, 21(1), Article 34. https://doi.org/10.1186/s13002-025-00782-4 *conducted by the Indigenous Peoples*

Sarmiento, F. O., Bush, M. B., McMichael, C. N. H., Chávez, C. R., Cruz, J. F., Rivas-Torres, G., Kavoori, A., Weatherford, J., & Hunt, C. A. 2024 Ecological Legacies and Ethnotourism:

Bridging Science and Community in Ecuador’s Amazonia. *Sustainability,* 16(11), 4664. https://doi.org/10.3390/su16114664

SERNANP. 2010. Diagnóstico integrado. Consultoría sobre Elaboración del Plan Maestro del Santuario Nacional Pampa Hermosa. Proyecto: “*Conservación de la diversidad biológica de las áreas naturales protegidas para la mitigación y adaptación al cambio climático*”. PRFNP-C-CON-007-2010-MACC II. SERNANP. Lima Perú. 220 p.

Silva Monterrey, N, A. Rodríguez & H. Castellanos con comunidades indigenas ye'kwana y sanema de la cuenca del Caura 2012 *Pautas para el manejo de los hábitats ye’kwana y sanema en la cuenca del Caura*. Organización Indígena de la Cuenca del Caura "KUYUJANI", La Universidad Nacional Experimental de Guayana-Centro de Investigaciones Antropológicas de Guayana,Forest Peoples Program. 67 p.

Taylor, L.P., Cronkleton, P., Barry, D., Stone-Jovicich, S. y Schmink, M. 2008. *Si lo vieras con mis ojos: Investigación colaborativa y cooperación con comunidades administradoras de bosques en Centroamérica*. CIFOR. Bogor, Indonesia. 47 p.

Vásquez-Fernández A, M Shuñaqui Sangama, Cash Ahenakew, Miriam Pérez Pinedo, Raúl Sebastián Lizardo, Judith Canayo Otto & Robert A. Kozak 2021 From “mutual respect” to

"intercultural respect": collaborating with Asheninka and Yine Peoples in the Peruvian Amazon, *The Journal of Legal Pluralism and Uno^icial Law*, 53:1, 127-153, DOI:

10.1080/07329113.2021.1889791

Venter A. y Breen C. 1998. Partnership Forum Framework: Participative Framework for Protected Area Outreach. *Environmental Management* 22: 803-815.

Vitaloni, M., Vargas, I., Eguiguren, P., Mogollón, A., Samico, I., López, J., Amarilla, D., Bertolotto, F., & Vazquez, M. 2018 Using participatory action research to improve care coordination in Latin America healthcare networks. *European Journal of Public Health*,

28(suppl_4). https://doi.org/10.1093/eurpub/cky213.220

Wallerstein, N., Duran, B., Oetzel, J. G., & Minkler, M. (eds.) 2018 *Community-based participatory research for health : advancing social and health equity* (Third edition.). Jossey-Bass, a Wiley Brand.

Zent, S., Zent, E. 2023 Collaborative Action Research for Biocultural Heritage Conservation. In: Rozzi, R., Tauro, A., Avriel-Avni, N., Wright, T., May Jr., R.H. (eds) *Field Environmental Philosophy. Ecology and Ethics*, vol 5. Springer, Cham.

Zent, S., Zent, E. 2023 Collaborative Action Research with the Jotï in Venezuela: Experiences in Autoethnography and TEK Vitality Assessment. In: Rozzi, R., Tauro, A., Avriel-Avni, N., Wright, T., May Jr., R.H. (eds) *Field Environmental Philosophy. Ecology and Ethics*, vol 5. Springer, Cham.
